# Hyperandrogenism and Hypokalemic Thyrotoxic Periodic Paralysis in a North American Adolescent Girl

**DOI:** 10.1210/jcemcr/luae083

**Published:** 2024-05-20

**Authors:** Anne Gladding, Joseph Bartoletti, Pallavi Iyer, Elizabeth Dabrowski

**Affiliations:** Department of Pediatrics, Division of Endocrinology and Diabetes, Medical College of Wisconsin, Milwaukee, WI 53226, USA; Department of Pediatrics, Division of Emergency Medicine, University of California San Diego (UCSD), San Diego, CA 92103, USA; Department of Pediatrics, Division of Endocrinology and Diabetes, Medical College of Wisconsin, Milwaukee, WI 53226, USA; Department of Pediatrics, Division of Endocrinology and Diabetes, Medical College of Wisconsin, Milwaukee, WI 53226, USA

**Keywords:** Graves disease, thyrotoxic periodic paralysis, hyperandrogenism

## Abstract

We present a unique case of hypokalemic thyrotoxic periodic paralysis (TPP) in an adolescent girl in North America. TPP is a rare but dangerous complication seen in thyrotoxic patients characterized by hypokalemia and acute proximal symmetric lower-extremity weakness. It is an especially rare phenomenon in pediatrics, with roughly 20 case reports described in adolescents worldwide; the majority are male. Our patient is a 14-year-old Asian girl with biochemical hyperandrogenism and known Graves disease who presented with an acute episode of lower-extremity weakness after eating a carbohydrate-rich meal. Laboratory workup revealed hypokalemia, hypomagnesemia, an undetectable thyrotropin, and hyperthyroxinemia. Electrolyte derangements responded well to supplementation, and the muscle weakness resolved with electrolyte normalization. Following improvement in thyroid function, the patient underwent thyroidectomy for definitive management of Graves disease. As TPP is potentially exacerbated by higher androgen and insulin levels, we suspect that with increasing rates of obesity and polycystic ovary syndrome, the incidence of TPP among adolescents may increase. It is therefore critically important that there is awareness and recognition of this serious diagnosis among all health care providers.

## Introduction

The exact incidence of thyrotoxic periodic paralysis (TPP) is unknown but some sources estimate occurrence in 1.8% to 1.9% of adult Asian patients with thyrotoxicity [1‐3]. There is a lower incidence in pediatric and non-Asian ethnic populations. Given its rarity, and that other types of periodic paralysis present in a similar fashion, TPP may be overlooked or misdiagnosed. Accurate diagnosis is challenging without a known history of hyperthyroidism, as is often the case, and clinicians should be alert to other symptoms in the patient's history such as weight loss, tachycardia, inattention, sleeplessness, or agitation that would indicate hyperthyroidism. While the most common cause of thyrotoxicosis is Graves disease, any cause of thyrotoxicosis can lead to TPP, including toxic nodular goiter, exogenous thyroxine use, and thyroiditis, among other entities [4]. Given its potential for progression to life-threatening emergencies such as fatal dysrhythmias, TPP should remain in the differential in all patients with muscle weakness and hypokalemia, particularly if these other factors are present in the history [5, 6].

**Figure 1.**
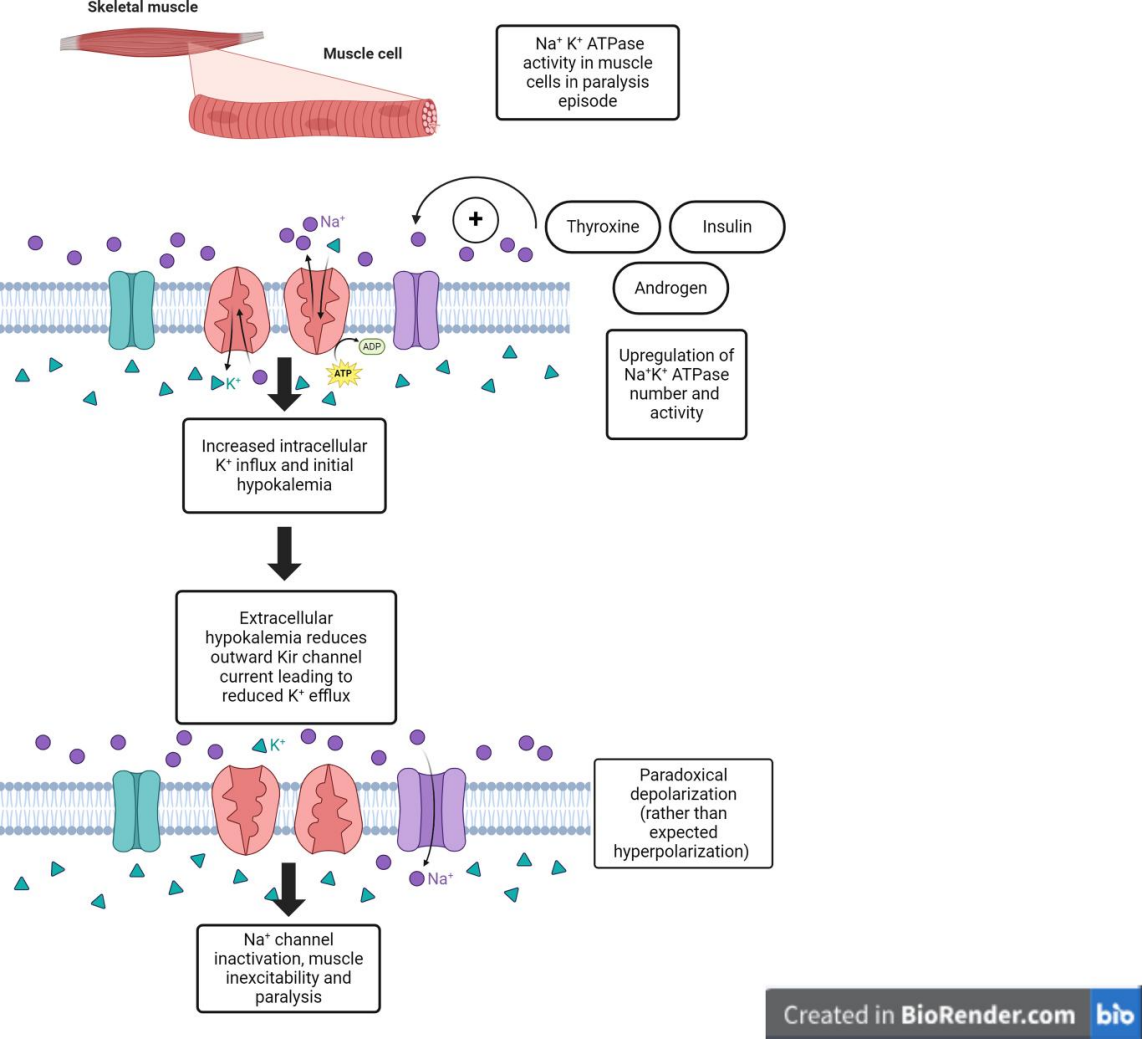
Proposed mechanism of thyrotoxic periodic paralysis (TPP). Various hormones including thyroxine, insulin, and androgens have direct and indirect effects to increase Na^+^-K^+^ ATPase channel activity in muscle cells. This enhanced activity results in increased intracellular K^+^ influx and creates an initial hypokalemia extracellularly. Normally, the increased Na^+^-K^+^ ATPase activity would be offset by increased K^+^ efflux via Kir channels. However, in TPP we see reduced K^+^ channel efflux leading to a paradoxical depolarization instead of the predicted hyperpolarization. The reduced K^+^ efflux may be caused by the initial hypokalemia itself (in muscle hypokalemia will decrease normally hyperpolarizing currents), a complete loss of function mutation in the Kir channel, or a hormone-mediated inhibition (eg, insulin) of the channel. The depolarization inactivates Na^+^ channels, resulting in muscle inexcitability and paralysis.

The underlying cellular mechanism of hypokalemia in TPP is not clearly known but researchers hypothesize that elevated thyroid hormone levels lead to increased activity of the Na^+^-K^+^ ATPase pump causing rapid intracellular potassium shifts [7]. Additional hormones including insulin, testosterone, aldosterone, and catecholamines are also known to upregulate Na^+^-K^+^ ATPase pump activity [7, 8]. The changes in the activity of the Na^+^-K^+^ ATPase pump from circulating hormones alone, however, do not completely explain the muscle paralysis in an acute TPP exacerbation. More recent findings show that loss-of-function mutations in *KCNJ18*, encoding Kir2.6, a skeletal muscle-specific Kir channel, result in decreased total potassium efflux and contribute to this phenomenon [7, 9]. The reduced potassium efflux may also be due to the initial hypokalemia itself or direct hormone-mediated inhibition of Kir channels. This creates a persistent, paradoxical depolarization of muscles with inactivated sodium channels and inhibits muscle excitability, leading to paralysis in TPP (Fig. 1).

Although hyperthyroidism is more common in female patients, TPP is much more common in males [10, 11]. To the best of our knowledge, there is only one other report of TPP in an adolescent female worldwide, reported in Korea [8, 12]. We present an adolescent girl in North America with Graves disease complicated by TPP and explore the potential mechanisms that placed her at an increased risk for developing TPP.

## Case Presentation

A 14-year-old Asian American girl presented to the emergency department with an acute episode of lower-extremity weakness after eating a carbohydrate-rich meal. Two months prior she had been diagnosed with Graves disease after undergoing workup for unintentional weight loss of 22.7 kg, increased anxiety, tachycardia, and poor sleep over the preceding year, which was initially attributed to stress with a return to in-person school after the COVID-19 pandemic. Laboratory evaluation revealed undetectable thyrotropin (TSH) less than 0.01 μIU/mL (normal reference range: 0.27-4.20 μIU/mL), elevated free thyroxine (T4) greater than 7.7 ng/dL (>99.1 pmol/L) (normal reference range: 0.9-1.7 ng/dL; 11.6-21.9 pmol/L), and elevated thyroid-stimulating immunoglobulin of 15.10 IU/L (normal reference range: ≤0.54 IU/L). She was prescribed methimazole 30 mg daily (0.44 mg/kg/d) and propranolol 10 mg twice daily (0.3 mg/kg/d).

In the emergency department, the patient reported a history of falls, tripping, and clumsiness preceding her diagnosis of Graves disease without any episodes of complete paralysis. There were 2 falls described the day of presentation—the first she quickly recovered from; however, with the second, she was rendered completely immobile, prompting urgent evaluation. Her clinical history was also significant for oligomenorrhea, with last spontaneous menstrual cycle reported more than 3 months prior to presentation and menarche more than 2 years prior at age 11 years. Notably, there was no family history of paralysis.

## Diagnostic Assessment

On presentation, the patient’s height was 154.9 cm, weight was 68 kg, and body mass index was 28.3. Her blood pressure was 164/73 mm Hg, temperature 36.3 °C and respiratory rate 36 breaths/minute. She was tachycardic with heart rate 130 beats per minute. Her initial physical examination demonstrated bilateral lower-extremity weakness, graded as 4/5 against gravity, and present but diminished patellar deep-tendon reflexes. She demonstrated normal upper-extremity strength and range of motion. There were no cranial nerve or sensory deficits. Extraocular movements were intact without nystagmus; she had normal phonation and no respiratory distress or dysphagia. Her electrocardiogram was significant for tachycardia and prolonged QTC of 571 ms (normal ≤ 446 ms). A computed tomography scan of the head showed no acute intracranial process. Laboratory workup revealed hypokalemia with potassium 2.7 mmol/L (normal reference range: 3.5-5.0 mmol/L), low magnesium 1.5 mg/dL (0.6 mmol/L) (normal reference range: 1.6-2.6 mg/dL; 0.7-1.1 mmol/L), a low TSH 0.01 mIU/L (normal reference range: 0.3-4.2 mIU/L) and an elevated free T4 4.1 ng/dL (52.8 pmol/L) (normal reference range: 0.7-2.0 ng/dL; 9.0-25.7 pmol/L), consistent with thyrotoxicosis (Table 1). Serum creatinine kinase, erythrocyte sedimentation rate, C- reactive protein, liver function tests, and a respiratory viral panel were normal. There was no leukocytosis on complete blood count. Serum alcohol, salicylate, and acetaminophen levels were undetectable. Her urine drug screen was negative.

**Table 1.** Biochemistry results on emergency department presentation

Test name	Initial presentation	Reference range
Sodium	142 mEq/L	136-145 mEq/L
	(142 mmol/L)	(136-145 mmol/L)
Potassium	**2.7** **mEq/L**	3.5-5.0 mEq/L
	**(2.7 mmol/L)**	(3.5-5.0 mmol/L)
Chloride	**110** **mEq/L**	98-107 mEq/L
	**(110 mmol/L)**	(98-107 mmol/L)
Bicarbonate	24 mEq/L	21-31 mEq/L
	(24 mmol/L)	(21-31 mmol/L)
Creatinine	**0.28 mg/dL**	0.50-0.80 mg/dL
	**(24.8 µmol/L)**	(44.2-70.7 µmol/L)
Magnesium	**1.5 mg/dL**	1.6-2.6 mg/dL
	**(0.6 mmol/L)**	(0.6-1.1 mmol/L)

Abnormal values are shown in bold font. Values in parenthesis are International System of Units.

## Treatment

The patient received oral potassium (40 mEq), intravenous magnesium (1 g), and propranolol (10 mg) and was transferred to our pediatric intensive care unit for further evaluation and management. Electrolyte abnormalities normalized with supplementation and laboratory values were monitored closely throughout her admission (Table 2). Muscle weakness improved following normalization of electrolyte abnormalities. Hyperthyroidism improved prior to discharge (Table 3) with administration of Lugol's potassium iodide-iodine solution (10 drops 3 times daily) and increased methimazole dose of 35 mg daily (0.5 mg/kg/d). She also continued propranolol 10 mg twice daily (0.3 mg/kg/d).

**Table 2.** Potassium and magnesium trends with replacement

Test name	Admission	Day 1	Day 2	48 h post discharge	Reference range
Potassium	4.0 mEq/L	4.2 mEq/L	3.9 mEq/L	4.0 mEq/L	3.5-5.0 mEq/L
	(4.0 mmol/L)	(4.2 mmol/L)	(3.9 mmol/L)	(4.0 mmol/L)	(3.5-5.0 mmol/L)
Magnesium	1.8 mg/dL	**1.7 mg/dL**	**1.6 mg/dL**	**1.7 mg/dL**	1.8-3.0 mg/dL
	(0.7 mmol/L)	**(0.7 mmol/L)**	**(0.6 mmol/L)**	**(0.7 mmol/L)**	(0.6-1.1 mmol/L)

Abnormal values are shown in bold font. Values in parenthesis are International System of Units.

**Table 3.** Thyroid biochemistry results including trend following discharge

Test name	ED value	Admission	Day 2 admit	48 h post discharge	Reference range
TSH	**0.01** **mIU/L**				0.3-4.2 mIU/L
	**(0.01 IU/L)**				(0.3-4.2 IU/L)
Free T4	**4.1 ng/dL**	**6.3 ng/dL**	**5.0 ng/dL**	**2.8 ng/dL**	0.7-2.0 ng/dL
	**(52.8 pmol/L)**	**(81.1 pmol/L)**	**(64.4 pmol/L)**	**(36.0 pmol/L)**	(9.0-25.7 pmol/L)
Total T3		**755 ng/dL**	**481 ng/dL**	**279 ng/dL**	100-210 ng/dL
		**(11.6 nmol/L)**	**(7.4 nmol/L)**	**(4.3 nmol/L)**	(1.5-3.2 nmol/L)

Abnormal values are shown in bold font. Values in parenthesis are International System of Units.

Abbreviations: ED, emergency department; T3, 3,5,3′-triiodothyronine; T4, thyroxine; TSH, thyrotropin.

## Outcome and Follow-Up

Following hospital discharge, our patient did experience residual lower-extremity weakness, particularly with certain activities, like climbing stairs at school. However, she never experienced a recurrence of a fall or paralysis. Given the severity of her presentation and a history of inconsistent medication adherence, definitive treatment of Graves disease was discussed with the family, and she underwent total thyroidectomy approximately 3 months following hospitalization for TPP. Postoperatively, she continues to do well on levothyroxine replacement and has not had any further episodes of weakness or muscle paralysis.

She also met the criteria for polycystic ovary syndrome (PCOS) based on the 2020 adolescent polycystic ovary syndrome guidelines published by Peña et al [13]. She has both irregular menstrual cycles defined according to years post menarche (>90 days for any 1 cycle >1-year post menarche) and biochemical hyperandrogenism with an elevated free testosterone concentration (Table 4). Her additional workup included a normal prolactin level, negative pregnancy tests, and normal DHEA-SO_4_ (dehydroepiandrosterone sulfate; see Table 4). We do not have any clinical evidence of nonclassic congenital adrenal hyperplasia; therefore, 17-hydroxyprogesterone levels were not obtained. She is not currently on medical therapy for PCOS but is implementing healthy lifestyle changes.

**Table 4.** Measured androgens

Test name	Result	Normal range
DHEA-SO_4_	125 μg/dL	37-307 μg/dL
	(3401.4 nmol/L)	(1006.8-8353.8 nmol/L)
Testosterone total, LCMS	**122 ng/dL**	< or = 40 ng/dL
	**(4.2 nmol/L)**	(< or = 1.4 nmol/L)
Testosterone free	**5.1 pg/mL**	0.5-3.9 pg/mL
	**(17.7 pmol/L)**	(1.7-13.5 pmol/L)

Abnormal values are shown in bold font. Values in parenthesis are International System of Units.

Abbreviations: DHEA-SO_4_, dehydroepiandrosterone sulfate; LCMS, liquid chromatography–mass spectrometry.

## Discussion

We present a unique case of TPP in an adolescent girl in North America. TPP presents with sudden onset of proximal muscle weakness secondary to intracellular shifts of potassium and decreased K^+^ efflux. These episodes are more common in the early morning and can be triggered by carbohydrate-rich meals, strenuous physical activity, medications such as glucocorticoids, or other physiologic stressors [14, 15].

Research suggests that several factors may contribute to electrolyte derangements and associated muscle paralysis. Extracellular potassium homeostasis is largely dependent on the skeletal muscles and is regulated by the interplay between sarcolemmal Na^+^-K^+^ ATPase pumps, which regulate the intracellular movement of potassium, and Kir channels, which regulate the extracellular movement of potassium. Increased levels of thyroid hormone upregulate the transcription of genes coding for the Na^+^-K^+^ ATPase pumps, increasing Na^+^-K^+^ ATPase pump activity by increasing sensitivity to circulating catecholamines [7, 16]. Moreover, in up to one-third of patients with TPP, loss-of-function mutations in *KCNJ8* encoding for Kir2.6 found in muscle cells may prevent the extracellular flow of potassium and exacerbate hypokalemia and muscle paralysis [17]. Similarly, insulin inhibits these channels, further reducing extracellular potassium, which may explain why the consumption of carbohydrate-rich foods often triggers an episode of muscle paralysis [18, 19].

The male predominance of TPP is still not entirely well understood, but the hypotheses are 2-fold. First, males have higher baseline levels of Na^+^-K^+^ ATPase in myoblasts compared to females, leading to increased intracellular influx of potassium at baseline. Second, androgens increase the expression and activity of Na^+^-K^+^ ATPase, further increasing potassium intracellular influx [20, 21]. In patients with normal function of *KCNJ8*, it is likely a combination of elevated insulin and androgen levels in addition to hyperthyroidism that leads to an acute episode of TPP.

Interestingly, our patient meets the criteria for PCOS with confirmed biochemical hyperandrogenemia and has evidence of insulin resistance with acanthosis nigricans. In addition to PCOS and acanthosis nigricans, her obesity worsens hyperinsulinemia and these factors combined likely led to her increased risk of developing TPP [22]. We hypothesize that rates of TPP in adolescent girls with Graves disease may increase given the obesity epidemic in North America and the increased prevalence of PCOS. Therefore, it will be prudent to check thyroid function tests in adolescent patients with obesity and hyperandrogenism presenting with paralysis and hypokalemia.

The goals of TPP management include normalization of electrolytes and treating the underlying cause of thyrotoxicosis to prevent future episodes of paralysis. Rapid detection and correction are important to prevent fatal dysrhythmias and respiratory failure, which have been reported as a cause of death in TPP [5, 6]. Patients may require monitoring for the risk of rebound hyperkalemia and hyperphosphatemia during the recovery phase. Since electrolytes and muscle strength normalize between episodes, prophylactic electrolyte supplementation is not beneficial in patients [16].

Although this clinical condition is rare in children and even more so in girls, TPP has potential lethal consequences if misdiagnosed and should be considered, especially in adolescent girls with Graves disease, insulin resistance, and hyperandrogenism who present with extremity weakness. As the prevalence of childhood obesity increases in North America with a resultant increased risk of comorbidities such as insulin resistance and hyperandrogenism, we suspect that the incidence of TPP may also rise in North America. Increased recognition of this condition, its mechanisms, and swift treatment will allow clinicians to prevent potential lethal complications in these children.

## Learning Points

- Although rare, TPP should be included in the differential diagnoses of patients presenting with episodic paralysis regardless of age or sex as paralysis often precedes the diagnosis of Graves disease.
- Clinicians should be aware of the life-threatening nature of TPP and urgent need to normalize electrolytes to prevent cardiac arrythmia.
- As rates of pediatric obesity and PCOS increase, TPP may become more common in pediatric patients and those with other risk factors, such as Asian heritage or uncontrolled hyperthyroidism. These patients should be counseled on presenting signs and symptoms of this serious condition.

## Data Availability

Data sharing is not applicable to this article as no data sets were generated or analyzed during the current study.

## Abbreviations

PCOS: polycystic ovary syndrome
T4: thyroxine
TPP: thyrotoxic periodic paralysis
CAH: congenital adrenal hyperplasia
